# Early Proteome Shift and Serum Bioactivity Precede Diesel Exhaust-induced Impairment of Cardiovascular Recovery in Spontaneously Hypertensive Rats

**DOI:** 10.1038/s41598-019-43339-8

**Published:** 2019-05-03

**Authors:** Leslie C. Thompson, Jonathan H. Shannahan, Christina M. Perez, Najwa Haykal-Coates, Charly King, Mehdi S. Hazari, Jared M. Brown, Aimen K. Farraj

**Affiliations:** 1 Environmental Public Health Division, U.S. Environmental Protection Agency, Research Triangle Park, NC USA; 2 School of Health Sciences, Purdue University, West Lafayette, IN USA; 3 College of the Mainland, Texas City, TX USA; 4 Department of Pharmaceutical Sciences, University of Colorado Anschutz Medical Campus, Aurora, CO USA

**Keywords:** Cardiovascular biology, Biomarkers

## Abstract

Single circulating factors are often investigated to explain air pollution-induced cardiovascular dysfunction, yet broader examinations of the identity and bioactivity of the entire circulating milieu remain understudied. The purpose of this study was to determine if exposure-induced cardiovascular dysfunction can be coupled with alterations in both serum bioactivity and the circulating proteome. Two cohorts of Spontaneously Hypertensive Rats (SHRs) were exposed to 150 or 500 μg/m^3^ diesel exhaust (DE) or filtered air (FA). In Cohort 1, we collected serum 1 hour after exposure for proteomics analysis and bioactivity measurements in rat aortic endothelial cells (RAECs). In Cohort 2, we assessed left ventricular pressure (LVP) during stimulation and recovery from the sympathomimetic dobutamine HCl, one day after exposure. Serum from DE-exposed rats had significant changes in 66 serum proteins and caused decreased NOS activity and increased VCAM-1 expression in RAECs. While rats exposed to DE demonstrated increased heart rate at the start of LVP assessments, heart rate, systolic pressure, and double product fell below baseline in DE-exposed rats compared to FA during recovery from dobutamine, indicating dysregulation of post-exertional cardiovascular function. Taken together, a complex and bioactive circulating milieu may underlie air pollution-induced cardiovascular dysfunction.

## Introduction

The epidemiological and experimental databases are replete with evidence linking air-pollution-induced adverse cardiovascular health outcomes with increased levels of key circulating mediators^1–5^. While these associations are strong, there is not yet enough research detailing the systemic events following air pollution exposure to accurately account for the reported cardiovascular health outcomes. The lack of available data stems in part from the complexity of systemic responses, which involve release of multiple mediators from injured or activated airway and alveolar epithelial cells, vascular endothelial cells, and/or infiltrating monocytes and neutrophils^6–10^. Although some mechanistic studies have established roles for specific circulating markers, it is likely that air pollution causes more subtle shifts in the circulating milieu such that the consequent health effects cannot be completely stimulated by addition of any single circulating factor, or completely prevented by pharmacological blockade or gene knockout of said factor.

An emerging alternative applied in recent studies is the assessment of the bioactivity of serum or plasma collected from subjects after exposure. This bioactivity describes the potential for a suite of serum or plasma-bound factors to collectively alter cellular, tissue, and systemic organ function. For example, plasma collected from humans exposed to NO_2_ altered gene expression patterns in naïve human coronary artery endothelial cells^11^ and serum collected from mice after exposure to mixed combustion emissions or wood smoke altered vascular reactivity of aortic rings collected from unexposed mice^12^. These approaches are predicated on the assumption that *in vivo* responses will in part mirror those measured *ex vivo*. Furthermore, *in vitro* and *ex vivo* responses may provide clues relating to potential pathophysiology, as altered function in key cell types and tissues *in vivo* are hallmarks of cardiovascular disease. While changes in a subset of *in vitro* or *ex vivo* tissues may not definitively predict *in vivo* responses, the presence of a bioactive circulating milieu after exposure enhances the plausibility of systemic factors as drivers of end organ responses above associations with increases in systemic factors alone. To date, however, serum bioactivity studies have only examined functional responses in recipient cells/tissue and have not been combined with measures of cardiovascular function in donor subjects, nor has the content of circulating milieu been routinely interrogated by high content approaches.

**Figure 1:**
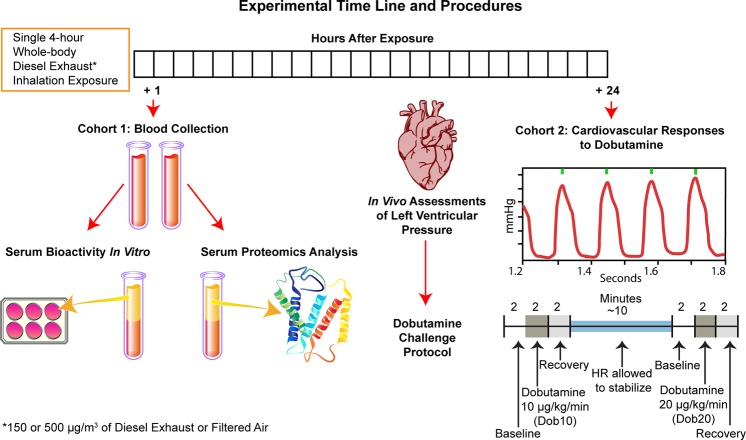
Experimental Time Line and Procedures. In order to establish that serum bioactivity and proteome changes occur in subjects that later present with cardiovascular dysfunction, SHRs were exposed to diesel exhaust at 150 or 500 µg/m^3^ or filtered air, and then split into two cohorts. Blood samples were collected from Cohort 1 one hour following exposure. Serum was used for proteomics analysis, or applied to endothelial cells *in vitro* to test for bioactivity. Twenty-four hours after exposure, SHRs from Cohort 2 were used for *in vivo* assessment of systemic cardiovascular responses to dobutamine stimulation and recovery while measuring left ventricular pressure (LVP) by pressure catheterization. LVP Data were recorded during a 2-minute baseline period, followed by 2 minutes of *i*.*v*. dobutamine at 10 µg/kg/minute, and a 2-minute recovery period. After allowing heart rates to stabilize for about 10 minutes, LVP data were recorded for another 2-minute period, 2 minutes of *i*.*v*. dobutamine at 20 µg/kg/minute, and a final 2-minute recovery period.

The purpose of this study was to determine if serum bioactivity, alterations in the circulating milieu, and cardiovascular dysfunction all take place in Spontaneously Hypertensive Rats (SHRs) after exposure to the same air pollution source. SHRs, which we have previously determined to be more sensitive to diesel exhaust (DE) exposure than their normotensive counterparts^13,14^, have well-documented high mean arterial pressure and left ventricular hypertrophy^15^. We hypothesized that exposure-induced impairment in cardiovascular function will be preceded by an altered circulating milieu that is bioactive *in vitro*. To that end, using a two-cohort design, SHRs were exposed once to DE, a source linked to near-road adverse clinical outcomes and a major contributor to traffic-derived fine particulate matter (PM_2.5_) and nitrogen oxide (NOx) emissions^16^. DE concentrations were targeted to 150 (DE150) or 500 (DE500) µg/m^3^ PM_2.5_ to mirror levels in congested urban areas at the low end^17^ and occupational exposure levels^18^ at the high end. In Cohort 1, bioactivity of serum from blood collected from SHRs 1 hour after the end of DE exposure was evaluated in rat aortic endothelial cells (RAECs), given that (1) systemic responses are triggered soon after exposure^19^, (2) endothelial cell changes strongly correlate with later adverse systemic cardiovascular responses^20,21^, and (3) endothelial cells interface directly with circulating factors (see experimental design in Fig. 1). Proteomic analysis was used to assess proteome-wide changes in the same serum samples as those used in the *in vitro* bioactivity assays. In Cohort 2, systemic cardiovascular function was interrogated in SHRs using a dobutamine stimulation and recovery challenge while measuring left ventricular pressure (LVP) by pressure catheterization, one day after exposure, consistent with the timing of DE-induced cardiovascular dysfunction reported in our previous study^22^. Finally, we integrate the findings to speculate on potential systemic mechanisms that drove the *in viv*o responses and suggest future studies to further elucidate mechanisms of action.

## Results

### Diesel exhaust characteristics and overt toxicity

Exposure chamber atmosphere properties and diesel exhaust characteristics are reported in Table 1. Filtered air (FA) chambers contained approximately 1 µg/m^3^ of PM_2.5_ compared to around 206 µg/m^3^ for the DE150 chambers and 509 µg/m^3^ in DE500 chambers. Particle diameters ranged from approximately 120–140 nm. Exposure chamber CO was around 1 part per million (ppm) in the FA group, 4 ppm in the DE150 group, and 10 ppm in the DE500 group. NO concentrations registered 0.1 ppm in the FA group, 1.5 ppm in the DE150 group, and around 5 ppm in the DE500 group, with similar concentrations for NOx. Chamber NO_2_ levels were low, ranging from 0 to 0.6 ppm and SO_2_ levels were below detectable limits. Relative humidity ranged from 35–55%, temperature ranged from 20–24 °C, and O_2_ percent was around 21%. No animals showed any signs or symptoms of overt toxicity to diesel exhaust exposure. Subject body mass for each exposure group are reported in Table 2.

**Table 1:** Particle Characterization and Chamber Conditions.

		FA	DE150	DE500
**Particle Characterization and Chamber Conditions**				
PM_2.5_	µg/m^3^	1.1 ± 0.6	206 ± 30	509 ± 51
MMAD	nm	—	124 ± 9	139 ± 13
GSD	nm	—	1.7 ± 0.0	1.6 ± 0.0
O_2_	%	21.0 ± 0.1	20.9 ± 0.1	20.8 ± 0.1
CO	ppm	1.0 ± 0.8	4.1 ± 0.0	10.1 ± 1.2
SO_2_	ppm	BDL	BDL	BDL
NO	ppm	0.1 ± 0.0	1.5 ± 0.1	4.9 ± 0.5
NO_2_	ppm	0.0 ± 0.1	0.1 ± 0.0	0.6 ± 0.1
NOx	ppm	0.2 ± 0.1	1.6 ± 0.2	5.5 ± 0.6
Temperature	°C	20.5 ± 0.4	24.3 ± 0.0	24.0 ± 1.0
Relative Humidity	%	54.3 ± 4.5	35.2 ± 0.2	38.7 ± 4.1
		(mean ± SD)		

Abbreviations – FA = Filtered Air, DE150 = 150 µg/m^3^ diesel exhaust, DE500 = 500 µg/m^3^ diesel exhaust, LVP = left ventricular pressure, BDL = below detectable limits, MMAD – mass median aerodynamic diameter, GSD – geometric standard deviation.

**Table 2:** Animal Weight.

			FA	DE150	DE500
**Animal Weight**					
Mass	g	Serum Cohort	309 ± 10	342 ± 27	312 ± 10
		LVP Cohort	308 ± 13	334 ± 41	307 ± 22
			(mean ± SD)		

Abbreviations – FA = Filtered Air, DE150 = 150 µg/m^3^ diesel exhaust, DE500 = 500 µg/m^3^ diesel exhaust, LVP = left ventricular pressure.

### Left ventricular heart rate and pressure data

**Figure 2:**
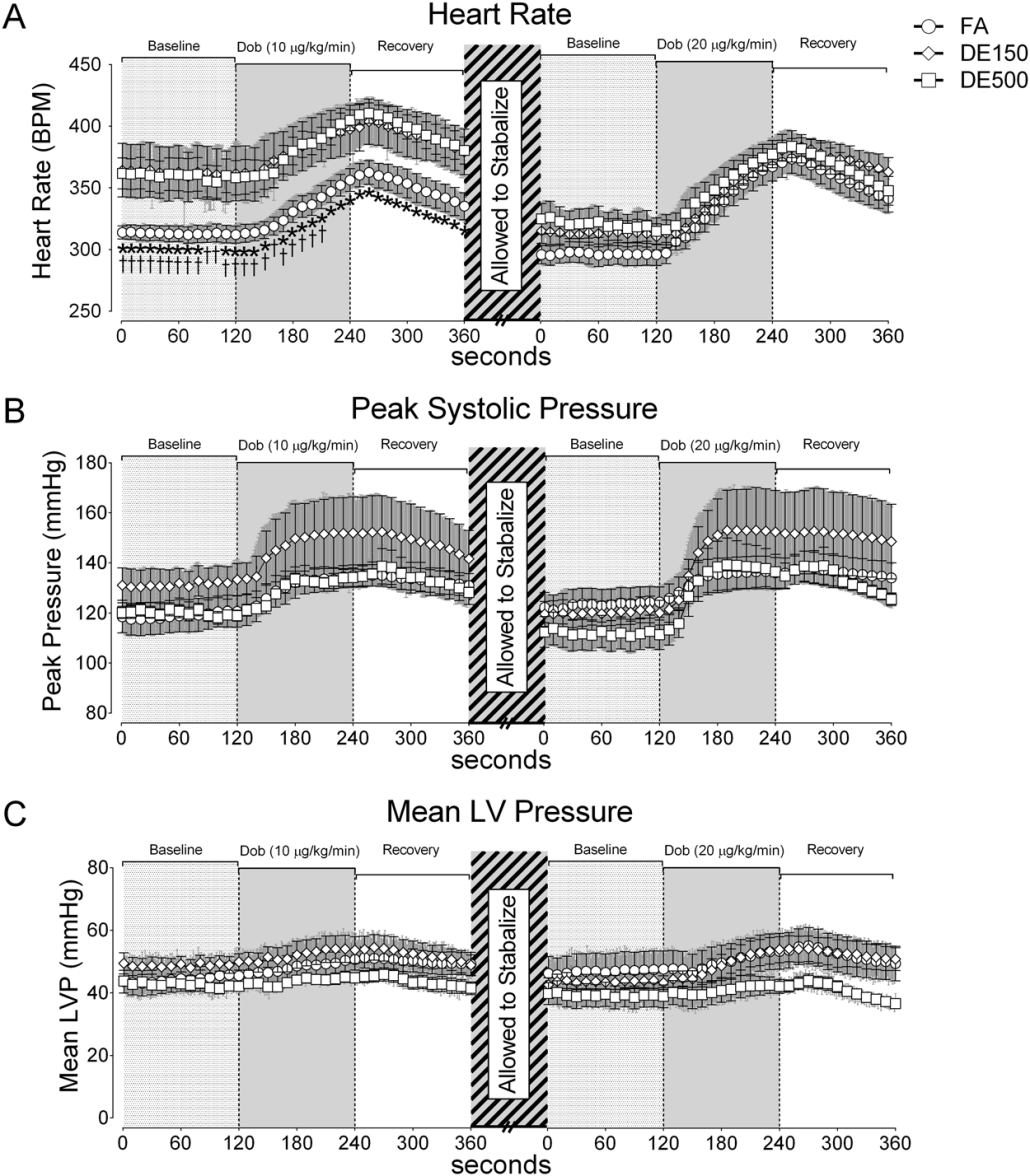
Left Ventricular Pressure Data. LVP was recorded using a pressure catheter one day after exposure. SHRs from Cohort 2 were used for *in vivo* assessment of cardiac function using a left ventricular pressure (LVP) catheter 24 hours after exposure. LVP Data were recorded during a 2-minute baseline period, followed by 2 minutes of *i*.*v*. dobutamine at 10 µg/kg/minute, and a 2-minutes recovery period. After allowing heart rates to stabilize for about 10 minutes, LVP data were recorded for another 2-minute period, 2 minutes of *i*.*v*. dobutamine at 20 µg/kg/minute, and a final 2-minute recovery period. (**A**) Heart rate data with both DE150 and DE500 significantly elevated over FA controls in the initial baseline period and into the first Dob10 challenge. (**B**) Peak systolic pressure was not different between groups. (**C**) Mean LV pressure began to drop in DE500 exposed SHRs during the final 2-minute dobutamine challenge (Dob20) and subsequent recovery period, diverging from the DE150 and FA controls, but was not statistically different from either. *For *p* < *0*.*05* DE500 vs. FA and †for *p* < *0*.*05* DE150 vs. FA as determined by repeated measures two-way ANOVA with Tukey’s post-test. n = 5–6.

Data collected from LV pressure (LVP) measurements are presented in Fig. 2 and Table 3. When baseline recordings were made after urethane anesthesia and placement of the LV catheter, heart rate in SHRs exposed to DE500 (*for *p* < *0*.*05*) and DE150 (†for *p* < *0*.*05*) were elevated compared to the FA group (Fig. 2A). Once heart rates stabilized after the first dobutamine recovery, there were no longer any significant differences between groups. We found no significant differences in LV peak systolic pressure (Fig. 2B) or mean LV pressure (Fig. 2C). However, mean LVP began to drop in DE500 exposed SHRs during Dob20 and subsequent recovery period, diverging from the DE150 and FA controls, though never reaching statistical significance. In Table 3, data are presented as mean ± SD, and rather than instance data, are presented as either the final baseline values, maximum dobutamine values, or final values at the end of the 2 minute recovery window, along with the associated statistical comparisons. The key differences between Fig. 2 and Table 3 is that in Table 3, the data for Mean Pressure were significantly lower in the DE500 group after the end of the recovery period following Dob20 when compared to both the FA group and the DE150 group. Also in Table 3, we reported Double Product (mmHg • HR in beats per minute *i*.*e*. BPM), which we found to be significantly elevated in the DE150 group compared to the FA group at the end of the baseline period and at the max value during Dob10.

**Table 3:** Left Ventricular Pressure Parameters by Time Period.

		group(n)	Baseline	Dob10 Max Val	After 2 min Rec	2^nd^ Baseline	Dob20 Max Val	After 2 min Rec
**Left Ventricular Pressure Parameters by Time Period**								
HR	BPM	FA(6)	312 ± 18	↑360 ± 24	↓307 ± 23	305 ± 27	↑398 ± 35	↓315 ± 22
		DE150(5)	364 ± 51 *	401 ± 44	↓347 ± 42	324 ± 30	↑414 ± 24	↓340 ± 40
		DE500(6)	357 ± 34 *	↑408 ± 21 *	↓346 ± 32	329 ± 34	↑413 ± 33	↓322 ± 33
Peak + dP/dT	mmHg/sec	FA(6)	8.2k ± 1.5k	↑15.6k ± 1.6k	↓7.9k ± 2.2k	7.8k ± 1.9k	↑18.0k ± 1.4k	↓8.2k ± 2.2k
		DE150(5)	10.4k ± 2.1k	↑17.9k ± 3.3k	↓9.8k ± 2.2k	7.6k ± 1.7k	↑18.4k ± 2.0k	↓8.7k ± 1.3
		DE500(6)	8.2k ± 2.1k	↑17.4k ± 2.2k	↓7.3k ± 1.5k	6.3k ± 1.6k	↑17.6k ± 1.9k	↓5.9k ± 1.5k
Peak -dT/dT	mmHg/sec	FA(6)	−7.6k ± 1.9k	−6.1k ± 0.7k	−7.8k ± 1.8k	−8.2k ± 1.9k	−6.4k ± 0.4k	↓−9.1k ± 2.3k
		DE150(5)	−8.9k ± 1.8k	−7.1k ± 0.8k	−8.9k ± 1.5k	−8.4k ± 1.2k	−6.9k ± 0.7k	↓−9.2k ± 1.6k
		DE500(6)	−7.8k ± 1.3k	−7.2k ± 0.7k	−8.1k ± 1.8k	−7.4k ± 1.6k	−6.8k ± 0.8k	−7.5k ± 1.6k
Peak SP	mmHg	FA(6)	121 ± 15	135 ± 10	122 ± 14	124 ± 14	137 ± 15	125 ± 13
		DE150(5)	135 ± 14	151 ± 32	↓129 ± 1	119 ± 11	↑152 ± 37	↓130 ± 12
		DE500(6)	119 ± 6	135 ± 10	117 ± 11	111 ± 14	↑135 ± 15	↓107 ± 8^†^
EDP	mmHg	FA(6)	11 ± 5	14 ± 6	14 ± 12	14 ± 14	14 ± 14	15 ± 14
		DE150(5)	5 ± 3	5 ± 3	4 ± 4	5 ± 4	7 ± 4	13 ± 12
		DE500(6)	3 ± 2	15 ± 28	5 ± 6	5 ± 6	9 ± 13	2 ± 4
Mean Pressure	mmHg	FA(6)	46 ± 10	51 ± 10	45 ± 12	48 ± 14	54 ± 12	48 ± 14
		DE150(5)	51 ± 5	54 ± 8	48 ± 3	42 ± 6	↑54 ± 9	50 ± 7
		DE500(6)	43 ± 4	45 ± 4	41 ± 8	39 ± 8	43 ± 5	35 ± 7 *†
Double Product	BPM•mmHg	FA(6)	37.8k ± 6.6k	↑48.7k ± 6.1k	↓37.5k ± 5.7k	38.0k ± 6.5k	↑54.5k ± 8.4k	↓39.4k ± 6.0k
		DE150(5)	49.5k ± 10.5k *	↑60.9k ± 17.0k*	↓44.8k ± 5.6k	38.5k ± 3.3k	↑62.6k ± 13.5k	↓44.2k ± 5.8k
		DE500(6)	42.5k ± 5.4k	↑54.9k ± 4.5k	↓40.6k ± 6.4k	36.8k ± 7.0k	↑56.1k ± 9.4k	↓34.6k ± 6.2k
		(Mean ± SD)						

Notes: FA = filtered air; DE150 = 150 µg/m^3^ diesel exhaust; DE500 = 500 µg/m^3^ diesel exhaust; Dob10 Max Val = maximum value during infusion with 10 µg/kg/min dobutamine; After 2 min Rec = value after 2 minutes of recovers; Dob20 Max Val = maximum value during infusion with 20 µg/kg/min dobutamine; HR = heart rate; BPM = beats per minute; dP/dT = change in pressure per change in time; Peak SP = peak systolic pressure; EDP = end diastolic pressure; Symbols for p < 0.05: *vs. FA; †vs. DE150; ↑ = increase vs previous data point; ↓ = decrease vs. previous data point.

### Responsive versus stable left ventricular pressure endpoints

Also provided in Table 3 is a within group, repeated measures analysis of each LVP parameter to determine: a) which parameters demonstrated relative stability across the baseline, dobutamine, and recovery test periods; and b) which parameters demonstrated greater responsiveness (*i*.*e*. statistically significant increases or decreases from the immediately previous time point) to dobutamine challenge and recovery. These results are marked for statistical significance (*p* < *0*.*05*) within each group using ↑, denoting a significant increase from the previous time point, or ↓, denoting a significant decrease from the previous time point. While all these functional parameters demonstrate some degree of responsiveness through baseline, dobutamine challenge, and recovery, the degree of change in some parameters should be more physiologically responsive to dobutamine/recovery (*e*.*g*. heart rate, peak positive pressure change *i*.*e*. +dP/dT, and double product) and some need to remain relatively stable in response to dobutamine and recovery (*e*.*g*. peak systolic pressure, end diastolic pressure, and mean pressure). Nonetheless, a few parameters that were responsive in the FA group showed less responsiveness in one or both DE group(s) and *vice versa*, i.e., parameters that were stable in the FA group showed less stability in one or both DE group(s). For example, heart rate was responsive in the FA group and DE500 group but showed to be less responsive in the DE150 group during Dob10. Similarly, peak dP/dT was responsive in the FA group and DE150 group after 2 minutes of recovery following Dob20, but peak dP/dT showed to be less responsiveness in the DE500 group. Peak systolic pressure in the LV showed statistical stability in the FA group but showed less stability in the DE150 and DE500 groups during the Dob20 and during recovery after Dob20. Mean LVP pressure was also stable in the FA and DE500 groups be showed less stability in the DE150 group during Dob20.

### Evidence of decreased postexertional heart function

**Figure 3:**
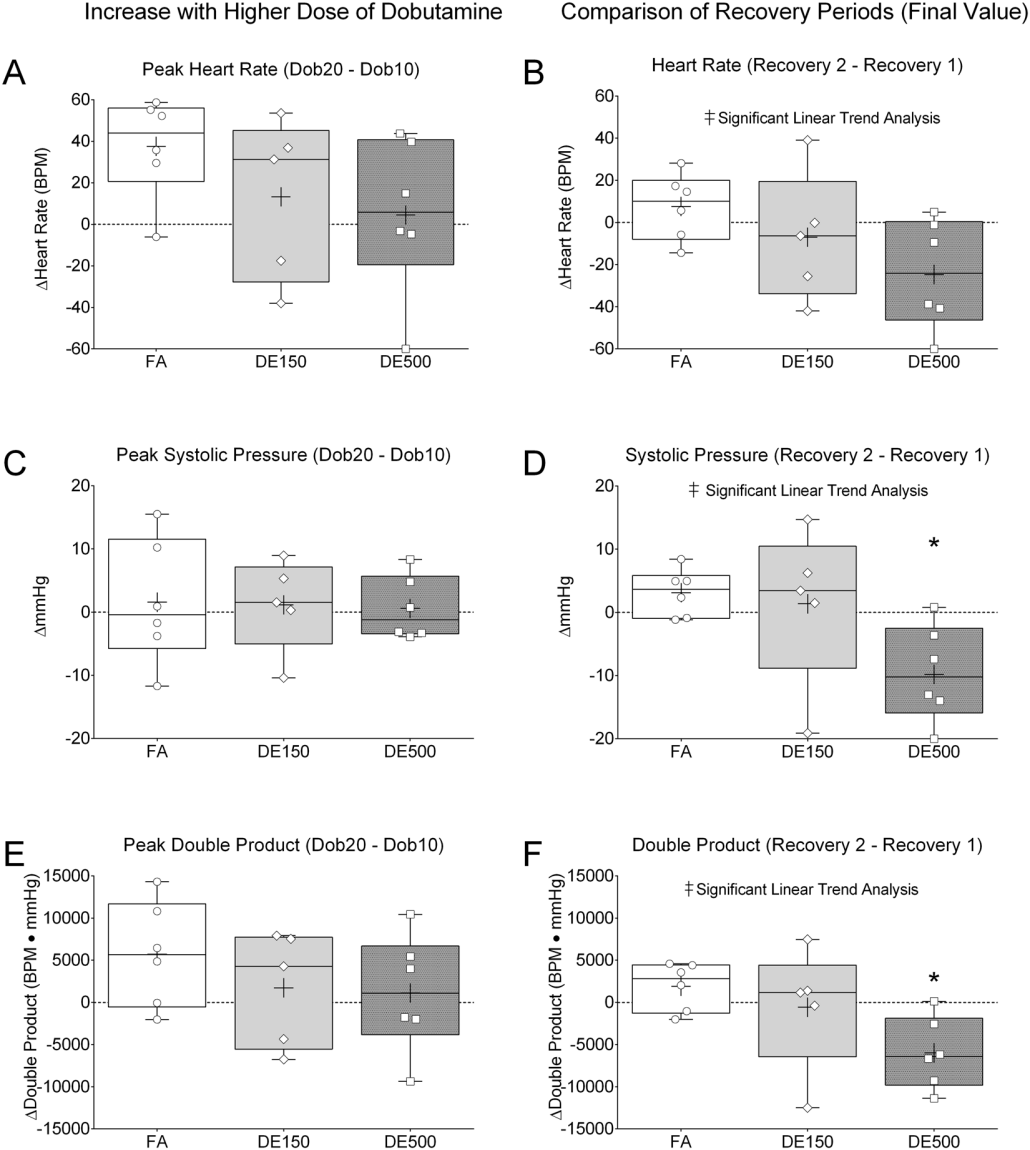
Comparison of Dobutamine Periods and Recovery Stability. We tested the differences between maximal responses between the two dobutamine periods and the stability of functional parameters during the recovery period. When we compared peak responses between Dob10 and Dob20 we found no differences between exposure groups for heart rate (**A**), systolic pressure (**C**), and Double Product (**E**). However, comparison of the values at the end of the recovery periods showed a decreasing linear trend (ANOVA) with DE concentration, with recovery 2 being lower than recovery 1 for heart rate (**B**), systolic pressure (**D**), and double product (**F**). Low values for recovery 2 vs. recovery 1 were significantly lower for DE500 than the FA group for systolic pressure (**D**) and double product (**F**). *For *p* < *0*.*05* DE500 vs. FA by one-way ANOVA and Tukey’s post-test. ^‡^for *p* < *0*.*05* linear trend analysis ANOVA. n = 5–6.

We went on to compare the differences in peak values from the first and second dobutamine challenges, as well as the differences in recovery values collected 2 minutes after the end of the first and second dobutamine challenges. Results of this analysis are presented in Fig. 3. We found no differences between groups when comparing the differences in peak heart rate (Fig. 3A), LV peak systolic pressure (Fig. 3C), and peak double product (Fig. 3E) derived by multiplying heart rate (BPM) by systolic pressure (mmHg). However, when comparing the final recovery values taken 2 minutes after the end of the first and second dobutamine challenges, heart rate demonstrated a significant decreasing linear trend (by ANOVA) from the FA group, to DE150, and then to DE500 (Fig. 3B). The comparison of final recovery values for LV peak systolic pressure also showed a significant decreasing linear trend across the 3 groups and the DE500 value was significantly lower than that of the FA group (Fig. 3D). Similarly, comparison of the double product values between the two recovery periods showed a significant decreasing linear trend and the value for the DE500 group was significantly lower than the FA group (Fig. 3F).

### Endothelial bioactivity of serum

**Figure 4:**
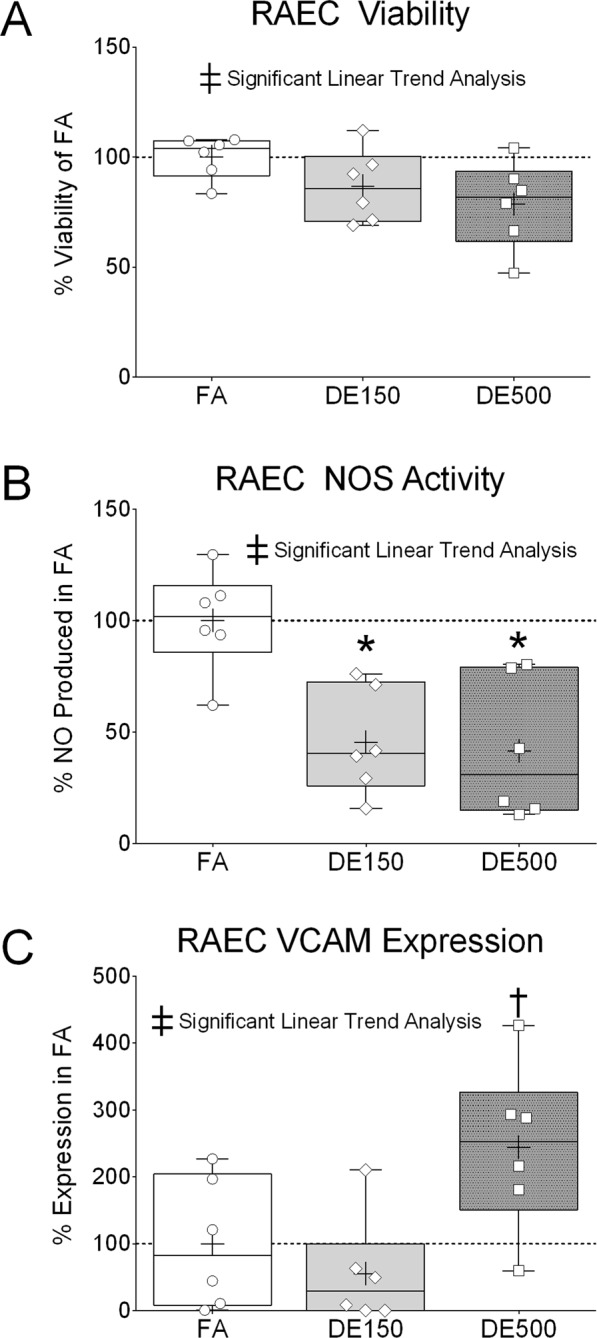
Endothelial Bioactivity of Serum. Rat aortic endothelial cells (RAECs) were treated *in vitro* with serum collected from FA or DE exposed SHRs. (**A**) RAEC viability was tested after 24 hours of serum treatment, which showed a significant decreasing linear trend (ANOVA) with increasing DE concentration. (**B**) RAEC nitric oxide synthase (NOS) activity was tested after 24 hours of serum treatment, which was significantly lower with DE exposure. (**C**) Vascular cell adhesion molecule-1 (VCAM-1) expression was tested after 3 hours of serum treatment, which showed a significant increasing linear trend (ANOVA) with increasing DE exposure, and was also significantly increased with DE500 serum treatment vs. DE150 serum treatment. *For *p* < *0*.*05* vs. FA and ^†^for *p* < *0*.*05* vs. DE150 by one-way ANOVA with Tukey’s post-test. ^‡^For p < 0.05 for linear trend analysis ANOVA. n = 6.

The results of *in vitro* treatment of rat aortic endothelial cells (RAECs) with serum collected from FA or DE exposed SHRs is presented in Fig. 4. Twenty-four hours after treatment of RAECs with serum, cell viability showed a significant decreasing linear trend (‡) with DE concentration but were not significantly different between groups by one-way ANOVA (Fig. 4A). However, nitric oxide synthase (NOS) activity was significantly decreased in RAECs treated with DE150 (*) or DE500 (*) serum for 24 hours as compared to FA serum (Fig. 4B), and a decreasing linear trend (‡). No statistically significant differences were found in *Alox15*, *Tbxas*, *Edn1*, *Ho1*, *Il33*, *Nos3*, *Icam1*, *Vcam1*, *Cxcl2*, and *Il6* mRNA expression in RAECs after 24-hour treatment with serum collected from exposed SHRs (see Supplemental Table S1). However, as shown in Supplemental Fig. S1A, *Alox15* expression was >2-fold downregulated relative to *Alox15*/*Gapdh* ratio in the FA and DE150 groups. In follow-up we tested 15-HETE concentrations in serum to see if a negative feedback system may explain any down-regulation of *Alox15* and found no differences between exposure groups (Supplemental Fig. S1B). After 3 hours of serum exposure, RAECs showed a significant increasing linear trend (‡) for cell surface vascular cell adhesion molecule-1 (VCAM-1) expression, which was significantly increased with serum treatment from DE500 exposed SHRs vs. treatment with serum from DE150 (†) exposed SHRs (Fig. 4C).

### Serum proteomics profiling

**Figure 5:**
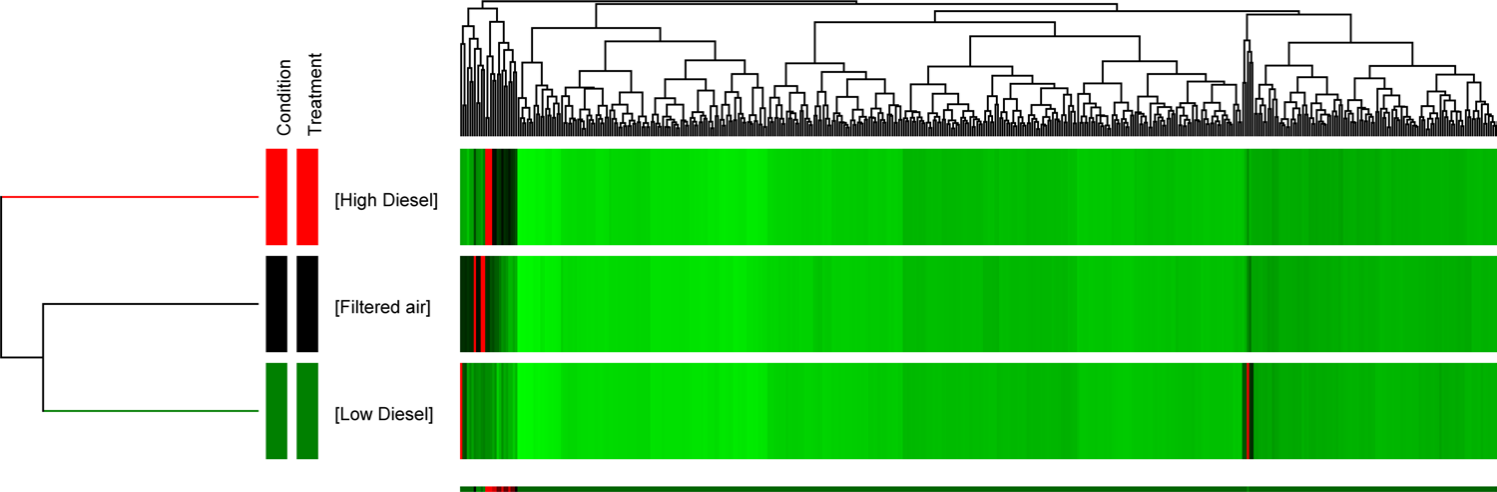
Hierarchical Clustering of Protein Data. Hierarchical clustering of serum proteins showed that the Filtered Air group and the DE150 group were more related than either group was to the DE500 group, indicated by the tree diagram to the left. The tree diagram at the top of the heat map indicates unsupervised hierarchical clustering of serum proteins using the Euclidian distance metric and Ward’s linkage rule for related proteins. Red in the heat map denotes proteins that were not detected, black denotes proteins of low abundance, and increasing intensity of green denotes proteins of higher abundance. Prior to clustering, peptides were screened using a one-way ANOVA (p < 0.05) with a Benjamini Hochburg multiple testing correction.

The analysis results of serum proteomics is displayed in Tables 4 and 5, and Fig. 5. The analysis of serum peptides uncovered 4116 total peptides, 3827 peptides were present in all replicates in at least one condition, *e*.*g*. FA, DE150, or DE500. Significance and fold change filters (*p* < *0*.*05*; >20% change) were used to determine if any peptides were different between groups. A one-way ANOVA utilizing Storey bootstrapping for multiple testing correction identified 375 peptides that were significantly altered (*p* < *0*.*05*), 186 of which showed a greater than 20% change between FA and DE500. The clustering pattern was confirmed by unsupervised hierarchical clustering of averaged replicate data (Fig. 5). Peptides were used to identify alterations in proteins following exposure to DE150, DE500, or FA (Table 4). Concentration-dependent alterations were observed in the number of proteins altered following exposure with most proteins being down-regulated (Table 4).

**Table 4:** Total Proteins Altered with DE Exposure.

	DE500 *vs*. FA	DE150 *vs*. FA	DE500 *vs*. DE150
**Total Proteins Altered with DE Exposure**			
Proteins	46	23	74
Up-Regulated	16	5	35
Down-Regulated	30	18	39
	Threshold determined by *p* < *0*.*05*		

Abbreviations: FA = filtered air; DE150 = 150 µg/m^3^ diesel exhaust; DE500 = 500 µg/m^3^ diesel exhaust.

Individual proteins were compared across groups to evaluate alterations due to DE exposures (Table 5). Many proteins altered following DE exposure were found to be related to the acute phase response, including those related to activation of the complement system (complement C1q subcomponent subunits A, B, and C, C4b-binding protein alpha chain, decorin, vitamin K-dependent protein S, and mannose-binding protein A), coagulation (factor IX), iron homeostasis (ceruloplasin, serotransferrin, and hemoglobin subunits alpha ½, beta 1, and beta 2), serine protease inhibitors (serine protease inhibitor A3N A3L, and A3K), and immune activation (interleukin-1 receptor accessory protein, forkhead box protein, receptor-type tyrosine-protein phosphatase V, IG kappa chain C region, A allele, Ig gamma-2A chain C region, and DNAJ homolog subfamily C member 27; Table 5). Other DE-modified proteins were involved in lipid metabolism (oxidized low-density lipoprotein receptor 1, apolipoprotein C-IV, and apolipoprotein C-III) as well as vascular permeability and tone (angiopoietin-2, Rho-associated protein kinase 1, T-kininogen, adenylate cyclase type 6, and profiling-1; Table 5).

**Table 5:** Proteomics Data.

Proteins Altered by *p* < *0*.*05*	*p-value*	Percent Change			Peptide#
		DE150 *vs*. FA	DE500 *vs*. FA	DE500 *vs*. DE150	
Coagulation factor IX (Fragment)	1.04e-5	−0.61	−13.21	−12.52	4
Heat shock 70 kDa protein 4	1.17e-5	4.85	−15.58	−21.18	1
Leucine-rich repeat-containing protein 8 C	1.92e-5	−5.69	−36.09	−28.77	1
Complement C1q subcomponent subunit A	2.44e-4	−3.00	−25.56	−21.90	1
Phosphatidylinositol-5-phosphate 4-kinase type-2 γ	4.50e-4	−4.16	−30.33	−25.13	1
Large proline-rich protein BAT3	5.58e-4	−3.10	24.54	28.40	1
Apolipoprotein C-IV	6.57e-4	−29.10	7.35	38.59	1
Oxidized low-density lipoprotein receptor 1	1.07e-3	−9.00	−2.20e5	−2.02e5	1
Protein kinase Cδ	1.21e-3	−23.03	−7.44	14.51	1
Serine protease inhibitor A3N	1.31e-3	−8.21	−18.47	−9.48	23
Rho-related GTP-binding protein RhoE	1.32e-3	0.32	16.22	15.86	1
Adenylate cyclase 6	1.54e-3	−3.39	31.40	35.86	1
Glutathione peroxidase 3	2.13e-3	−10.42	−2.65	7.57	7
Selenoprotein P	2.57e-3	−5.89	−20.12	−13.44	3
Ig κ chain C region, A allele	2.79e-3	62.41	−62.72	−164.27	2
Apolipoprotein A-II	2.97e-3	−24.17	−5.06	18.20	5
Calcineurin subunit B type 1	3.06e-3	−5.43	11.21	17.25	1
Serotransferrin	3.38e-3	−12.53	18.75	33.64	2
Complement C1q subcomponent subunit C	3.42e-3	−0.42	−20.17	−19.67	3
PHD finger protein 10	3.56e-3	5.92	−14.07	−20.82	1
Sodium- and chloride-dependent glycine transporter 1	3.64e-3	0.44	17.22	16.70	1
Centromere protein T	3.72e-3	−0.76	11.30	12.15	1
Angiopoietin-2	4.31e-3	5.16	−24.17	−30.57	1
Transmembrane protein 106 A	4.45e-3	7.28	−4.48	−12.08	1
Complement C1q subcomponent subunit B	4.59e-3	−2.61	−15.72	−12.78	2
Receptor-type tyrosine-protein phosphatase epsilon	5.16e-3	−47.16	22.09	79.67	1
Ig gamma-2A chain C region	5.74e-3	73.50	−46.42	−154.05	2
Hemoglobin subunit β-2	6.00e-3	95.66	103.09	3.80	3
Hemoglobin subunit α-1/2	6.15e-3	94.13	102.91	4.52	10
Hemoglobin subunit β-1	7.09e-3	93.76	95.63	0.96	14
Ceruloplasmin	7.92e-3	3.76	−18.13	−22.57	46
DnaJ homolog subfamily C member 27	8.03e-3	13.71	−14.14	−29.79	1
Forkhead box protein J1	8.43e-3	−4.93	−32.51	−26.29	1
Mannose-binding protein A	9.09e-3	−12.41	−15.45	−2.70	2
E3 ubiquitin-protein ligase UBR4	9.18e-3	−4.31	11.72	16.53	1
C4b-binding protein α chain	1.00e-2	−34.84	41.77	91.17	7
Apolipoprotein C-III	1.02e-2	−12.21	13.47	27.33	4
Rho-associated protein kinase 1	1.31e-2	8.59	−20.99	−31.38	2
Ephrin type-A receptor 3	1.32e-2	0.68	−10.28	−11.03	1
Tetratricopeptide repeat protein GNN	1.35e-2	−0.96	18.08	19.22	1
Serine protease inhibitor A3L	1.38e-2	7.38	−21.69	−30.67	5
Tetratricopeptide repeat protein 35	1.39e-2	1.19	−18.98	−20.40	1
BAG family molecular chaperone regulator 5	1.44e-2	3.47	−21.40	−25.61	1
Vomeronasal type-1 receptor A13	1.44e-2	0.66	13.34	12.60	1
Serine protease inhibitor A3K	1.59e-2	7.13	−17.28	−25.65	29
Uncharacterized protein C19orf44 homolog	1.62e-2	4.78	11.47	6.38	1
Peroxisomal acyl-coenzyme A oxidase 2	1.64e-2	4.45	17.59	12.58	1
Interleukin-1 receptor accessory protein	1.66e-2	3.00	−20.06	−23.66	1
Plectin	1.82e-2	6.18	−16.21	−23.39	2
Parvalbumin alpha	1.94e-2	−2.67	8.68	11.59	1
Transcription activator BRG1	2.02e-2	3.50	−8.17	−11.96	1
Receptor-type tyrosine-protein phosphatase V	2.16e-2	−0.59	−20.54	−19.84	1
Interferon-induced, double-strand RNA-activated protein kinase	2.18e-2	−0.50	10.28	10.83	1
T-kininogen 1	2.24e-2	4.66	−21.78	−27.45	19
Scm-like with four MBT domains protein 1	2.28e-2	−6.14	3.77	10.14	1
Decorin	2.35e-2	−13.70	−9.56	3.77	1
WD repeat-containing protein 44	2.50e-2	25.45	−10.27	−38.33	1
Trimeric intracellular cation channel A	2.51e-2	−3.20	−15.94	−12.34	1
Latrophilin-1	2.60e-2	−1.93	15.20	17.43	1
Profilin-1	2.62e-2	−2.31e4	−1.56	2.27e4	1
Probable G-protein coupled receptor 173	2.66e-2	−15.57	−4.91	10.17	1
Vitamin K-dependent protein S	2.68e-2	−9.23	2.61	12.08	1
Transient receptor potential cation channel M8	3.37e-2	9.07	−2.72	−12.04	1
Solute carrier family 12 member 8	4.00e-2	−4.57e3	4.15e3	1.98e5	1
DNA topoisomerase 1	4.14e-2	−1.19	16.36	17.75	1
Cyclin-L1	4.38e-2	−17.20	−0.33	16.81	1

## Discussion

This study provides evidence indicating that diesel exhaust (DE) exposure impacts serum bioactivity, the circulating proteome, and cardiovascular function in SHRs. More specifically, relative to SHRs exposed to filtered air, a single 4-hour diesel exhaust (DE) inhalation exposure resulted in impairment of postexertional cardiovascular function one day later during recovery from pharmacological challenge with the sympathomimetic dobutamine. The interrogation of serum collected from similarly-exposed SHRs 1-hour after exposure revealed enhanced bioactivity of serum in naïve rat aortic endothelial cells, predominantly by decreasing activity of NOS. Proteomic assessment of this serum indicated significant changes in the levels of 66 proteins related to vascular function, lipid metabolism, iron homeostasis, redox regulation, and the acute phase response. These findings indicate the need for more holistic examinations of the circulating milieu in air pollution-induced adverse cardiovascular health outcomes.

First, the exposure to DE in SHRs did lead to impairment of cardiovascular responses to dobutamine stimulation and recovery. The first indication of cardiovascular impact appeared after the onset of LVP assessments in anesthetized SHRs. Heart rates in both DE groups were elevated prior to administration of any dobutamine. Though we did not assess autonomic activity in the current study, this finding supports our previous study showing that DE inhalation in SHRs promotes sympathetic activation^22^. Interestingly, heart rates in the DE groups appeared to reset toward the level of the FA group after the first dobutamine challenge. Normally sympathetic stimulation (*i*.*e*. epinephrine/norepinephrine release) increases heart rate by increasing cellular cyclic AMP levels but it has been reported that dobutamine antagonizes *in vitro* production of cyclic AMP when administered with epinephrine^23^. The next indication of cardiovascular impact was that recovery values for HR, LVP, peak systolic pressure, and double product did not return to a stable baseline after the second of two dobutamine challenges. Impaired recovery after dobutamine in DE-exposed SHRs is consistent with impaired post-exercise recovery of cardiac function in humans exposed to air pollution^24^, which included double product, a correlate of oxygen uptake by the myocardium during exercise^25^. Low systolic pressure after exercise challenge (*i*.*e*. postexertional hypotension), often manifesting as syncope, is linked to increased risk for cardiac events^26^. Additionally, the absence of a reflex increase in heart rate in response to postexertional hypotension suggests impairment of arterial baroreflex control of heart rate^27^. Although we did not measure baroreflex responses in the present study, the loss of baroreflex sensitivity would be consistent with our previous findings that showed that acrolein inhalation reduced baroreceptor sensitivity in the same hypertensive rat strain used in the present study^28^. While the precise biological mechanisms that mediate these integrated cardiovascular responses to DE exposure are unclear, our proteomics assessment identified alterations in proteins related to vascular tone, smooth muscle contraction, and calcium transport. Many of the proteins seem to be intracellular proteins, which may indicate the presence of circulating microparticles (*i*.*e*. shed membrane vesicles) containing cellular constituents. Endothelial microparticles have been linked to several cardiovascular dysfunctions, potentially reducing the proper vascular response to fluctuations in hemodynamic pressure^29^, a physiological change evident in the results of our study.

Second, DE inhalation produced bioactive serum in blood collected from SHRs one hour after exposure. While the novelty of our study is the connection of all 3 events, *i*.*e*., changes in cardiovascular function, serum bioactivity, and serum proteome following DE exposure, it is important to note that the serum bioactivity in our study parallels that of other studies, specifically the decrease in NOS activity and increased VCAM-1 expression. This is also important because postexertional cardiovascular recovery, as we have just described, requires complex, integrated responses between the cardiovascular system and autonomic nervous system^30^, as well as a healthy, responsive population of vascular endothelial cells^31^. In this regard, NOS is a key indicator of vascular endothelial cell function and often investigated following DE exposure. For example, DE exposure has been linked to vascular endothelial NOS dysfunction after *in vivo* inhalation^32,33^ and by direct action of the DE particle components 1,2-naphthoquinone^34^ and phenanthraquinone^35^ when added to *ex vivo* isolated aortic segments. Moreover, loss of nitric oxide availability likely promotes increased VCAM-1 expression^36^, which leads to our *in vitro* examination of VCAM-1. We found that stimulation of RAECs *in vitro* with serum collected from SHRs exposed to DE500 caused upregulation of VCAM-1 expression when compared to treatment with serum collected from DE150. Although the difference in VCAM-1 expression between RAECs treated with serum from FA and RAECs treated with serum from DE500 was not statistically significant with a one-way ANOVA (P = 0.06), it was different from DE150 (see † in Fig. 4C) Also, analysis of all 3 means resulted in a statistically significant positive linear trend (see ‡ in Fig. 4C). This discrepancy could be due to timing as the VCAM assessment was after a 3-hour exposure to serum whereas the NOS data was derived after a 24-hour exposure. In any case, our finding is consistent with a report indicating upregulation of VCAM-1 in primary human coronary artery endothelial cells after 24-hour treatment with human plasma collected from DE exposed volunteers^11^. Cell culture media from DE particle-exposed airway epithelial cells produced similar increases in VCAM-1 in endothelial cells treated *in vitro*^37^. Circulating VCAM-1 was also reported to be slightly (but not significantly) elevated in a controlled human study after DE exposure^38^. RAEC viability after treatment with serum from DE-exposed SHRs was not significantly different from cells treated with serum from FA and DE150 groups, but there was a significant trend downward as DE exposure concentration increased, which could explain, in part, the presence of many intracellular proteins found in our proteomics data. Collectively, the *in vitro* findings point to a change in the endothelial bioactivity of serum after exposure to diesel exhaust indicating the potential to alter vascular tissue function *in vivo*. We suspect that loss of NOS activity and VCAM-1 induction following *in vitro* stimulation with serum from DE exposed SHRs demonstrates the potential for loss of precision responsiveness needed for optimal postexertional cardiovascular recovery *in vivo*.

Lastly, alterations in serum proteome identified one hour after DE exposure, especially those related to oxidative stress, could explain the modified endothelial and cardiovascular function. These include proteins involved in intracellular signaling in the cardiovascular system associated with pathophysiologic responses, e.g. adenylate cyclase, calcineurin, and RhoE^39,40^. The presence of these proteins in the serum may reflect cytotoxicity in vascular tissue caused by DE exposure, as takes place with oxidative stress^41^. Of the factors that increased in serum, hemoglobin subunits α-1/2, β-1, and β2 did so to the highest extent in both the DE500 and DE150 groups. Free hemoglobin undergoes autoxidation as it becomes deoxygenated and as hemoglobin tetramers breakdown into dimers^42^. Circulating free hemoglobin and associated reactive oxygen species tend to interfere with essential vascular functions by sequestering NO and transforming it to peroxynitrite^43^, which could be made worse following DE exposure. Conversely, heat shock protein 70 was decreased in serum from DE500 exposed SHRs. This stands in contrast to a report stating that heat shock protein 70 increases in the plasma of ApoE knockout mice after chronic exposure to DE. Heat shock proteins can function as chaperone proteins that help cells cope with oxidative stress and are generally regarded as protective^44^. However, in certain conditions such as metabolic syndrome, heat shock protein 70 (hsp70) decreases in the circulation^45^. Furthermore, heat shock protein 70 can be cleared or taken up by macrophages and other antigen presenting cells when it is complexed to damaged proteins^46,47^. While the net ramifications of all the proteins that increased or decreased after DE exposure remain unknown, it seems likely the alterations in the circulating proteome after DE exposure may set the stage for an array of negative impacts in the cardiovascular system.

In addition to the proteomic findings mentioned above, one key group of proteins consistently diminished by DE exposure are those related to the acute phase response. For example, interleukin-1 receptor accessory protein was decreased following DE500 exposure. This protein is involved in interleukin-1 signaling that initiates the systemic acute phase response^48^, including complement system activation and changes in coagulation, proteases, iron levels, and immune system homeostasis^49^. Along those lines, we also found that DE exposure reduced complement system proteins C1q subunits A, B, and C, and mannose-binding protein-A. C1q is a component of the C1 enzyme complex directly involved in complement system activation^50^, which has been reported following DE exposure^51^. Factor IX of the intrinsic coagulation pathway and serine protease inhibitors A3N, A3K, and A3L were also reduced following exposure to DE500. These findings are consistent with a previous report detailing a lack of evidence for acute phase response in the livers of mice following DE exposure at even higher concentrations than used in our study^52^. The absence or impairment of the acute phase response following DE inhalation may lead to a period of heightened susceptibility to infection, as is evident with malnutrition-related decreases in acute phase response^53^. In fact, increases in ambient PM_2.5_ have recently been linked to increased incidence of acute lower respiratory infections^54^. Thus, these reductions in acute phase response elements supports the idea that changes in circulating factors after air pollution exposure may diminish the capacity of an individual to handle subsequent physiological stressors.

Some limitations should be considered with this study. First, SHRs were anesthetized with sodium pentobarbital, so residual anesthetic likely remained in the serum used to treat RAECs given that the half-life of sodium pentobarbital is approximately 4 hours. Nonetheless, an anesthetic was required for high volume blood collection and alternative anesthetics are burdened by their own limitations. For example, isoflurane has been shown to interfere with endothelium-dependent vasodilation^55^ and ketamine/xylazine and tribromoethanol have been shown to induce endothelial injury more rapidly than sodium pentobarbital^56^. Additionally, the SHRs used as a model in this study collectively showed unexpected reductions in absolute value for peak diastolic dP/dT during dobutamine challenge in all groups. In our study, dobutamine infusion resulted in reduced absolute value for diastolic dP/dT in all groups. These findings may be explained by slower Ca^2+^ uptake in myocytes in SHRs^57^ such that excess myocyte free Ca^2+^ during dobutamine challenge could have impaired cardiac lusitropy. Another important note is that we have only examined the serum proteome and the resulting endothelial bioactivity at a single time point after exposure. It remains unclear when peak shifts in the proteome after DE exposure occur, as changes in circulating factors are likely to be dynamic over time. This limitation may be best demonstrated by the fact that decreased NOS activity, which may in fact be decreased NOS expression, in the DE150 group was not matched by an increase in VCAM-1 expression as was seen in the DE500 group. Furthermore, the RT-PCR data for endothelin-1 and VCAM were not in agreement with the NOS activity and VCAM protein data. While the precise reasons for these discrepancies are unclear, mRNA, protein expression, and protein activity do not always change to the same extent within a single window of time, especially when varying stimulated doses, in this case of DE, may impact to varying degrees the expression/degradation rate of mRNA and protein, and even post-translational protein modifications. Different stimuli may also initiate different rates of down-regulation followed by upregulation and *vice versa*. In the case of VCAM-1, NOS-independent pathways may have altered VCAM-1 expression. Finally, while a focus on systemic inflammation as a driver of the effects in this study may be plausible, specific markers of inflammation were not measured, thus limiting any linkage of the cardiovascular and endothelial responses to specific inflammatory pathways. We opted to carry-out a global proteomic assessment because of the uncertainty in the identification of the factors that may be driving such responses.

This study lays the groundwork for more mechanistic lines of inquiry in the future. For example, keying in on specific indicators of inflammation like interleukins 1β and 10, tumor necrosis factor, and C-reactive protein. Uncovering the time course of changes in VCAM-1 and NOS (*e*.*g*. NOS3 vs. NOS2 and expression vs. activity) may be very informative for understanding how DE exposure results in systemic cardiovascular dysfunction in SHRs. Utilizing the adoptive transfer approach on isolated aortic segments collected from naïve SHRs to test changes in vascular function may also be informative. Moreover, utilizing the adoptive transfer of serum from exposed rats to naïve rats to see if the systemic cardiovascular function can be replicated would be highly advantageous, provided complicating factors like how much serum needs to be transferred to successfully transfer and initiate the progression of cardiovascular dysfunction are properly addressed. Finally, exploring sex-specific responses may also help elucidate mechanism and issues of susceptibility.

In conclusion, this study enhances plausibility of the hypothesis that an array of changes in circulating factors after DE exposure can, in turn, precipitate measurable alterations in systemic cardiovascular function. While alterations in the circulating milieu were indeed complex, the findings in fact point to perturbation of various systemic pathways, including vascular function, lipid metabolism, iron homeostasis, redox regulation, and the acute phase response. Thus, it would be worthwhile to explore the impacts of antagonism of these pathways on the *in vivo* cardiovascular responses to diesel exhaust to definitively assign causality in future studies. Moreover, we provide evidence in the same model that air pollution-induced impairment of systemic cardiovascular function occurs in conjunction with changes in the circulating proteome and serum bioactivity. These findings are consistent with much of the data in epidemiological and clinical exposure studies that associate changes in systemic markers with adverse cardiovascular health outcomes. Still, the biological responses appear to consist of concurrent changes in systemic circulating factors and autonomic nervous system responses, which point to the complexity of air pollution health effects.

## Materials and Methods

### Ethical statement

All studies were carried out in accordance with the guidelines of, and approved by, the Institutional Animal Care and Use Committee at the U.S. EPA’s National Health and Environmental Effects Research Laboratory.

### Experimental design

We have previously shown the Spontaneously Hypertensive Rat (SHR) model to be more sensitive to diesel exhaust (DE) exposure than their normotensive counterparts^13,14^. We designed our current study to include two primary cohorts of SHRs (Fig. 1):

#### Blood Collection Cohort

SHRs were grouped into low or high concentration DE groups or a filtered air control group and underwent terminal blood collection one hour after exposure. Serum was isolated from the collected blood, which was used for two purposes:

- Rat aortic endothelial cells were cultured for 24 hours in media containing the serum (diluted to 10% in media) followed by subsequent testing of various *in vitro* markers of exposure
- Proteomic characterization

#### Left Ventricular Pressure (LVP) Cohort

SHRs underwent LVP assessments one day after exposure to low or high concentrations of DE or filtered air.

### Sample size analysis

The sample size analysis for this study was based on our previous study of DE exposure in heart failure prone rats in which we found that heart rates were different during exercise recovery^22^. In that study the effect size (d) was about 35 beats/minute with a SD in heart rate of approximately 20 beats/minute. Based on Cohen’s sample size calculations for effect size index (f = 0.5*d/SD) we calculated f = 0.875^58^. Sample size analysis was conducted using R Studio software (version 3.1.2) with the ‘pwr’ package (https://cran.r-project.org/web/packages/pwr/pwr.pdf) and ‘pwr.anova.test’ command. We set k = 3 for our two DE groups and 1 control group, f = 0.875, significance level = 0.05, power = 0.8, and solved for n. This yielded n = 5.33 so we set n = 6 for the study.

### Animals

We utilized two cohorts of SHRs (for LVP assessment and blood collection) each with 3 experimental groups containing 6 SHRs per group. Thus, thirty-six, 12-week old male SHRs were purchased from Charles River (SHR/NCrl, Strain Code 007, Raleigh, NC, USA). SHRs acclimated for at least one week in our Association for Assessment and Accreditation of Laboratory Animal Care-approved facility prior to any experimentation. They were housed in plastic cages (2/cage) and maintained on a 12-hour light/dark cycles at approximately 22 ± 1 °C and 50% relative humidity (RH). Food (Prolab RMH 3000; PMI Nutrition International, St Louis, MO) and water were provided *ad libitum*.

### Diesel exhaust exposure

SHRs were exposed to either FA, DE150, or DE500 for 4-hours, as previously described^22,59^. In short, DE originated from a single-cylinder 0.320 L displacement Yanmar L70 V diesel generator operated at 3600 rpm on low sulfur diesel fuel (16 ppm) at a 3-kW load and was diluted with high efficiency particulate air (HEPA)-filtered room air and delivered to exposure chambers. The FA group was exposed to HEPA-filtered room air in a separate exposure chamber. DE contained ultrafine mode PM and NO_2_ concentrations comparable to observations in United States and European traffic tunnels and roadways^60–62^.

### Left ventricular pressure 24-hours after exposure

One day after exposures, SHRs were anesthetized with urethane (1.5 mg/kg *i*.*p*., Sigma) and prepared for LVP measurement by right carotid arterial catheterization with a 2-French transducer (SPR-320, Millar Instruments, Houston, TX). The left jugular vein was cannulated for cardiac stress test by sympathomimetic infusion (dobutamine HCl). The pressure transducer was calibrated using a Pressure Control Unit (Model 2000, Millar Instruments) and connected to a data acquisition interface (Powerlab 4/30, ADInstruments, Dunedin, New Zealand) and computer recording data at 1000 Hz sample rate. The probe was advanced into the LV and subjects were allowed to rest for 3–5 minutes before making a 2-minute baseline recording. Then freshly diluted dobutamine HCl (dissolved in 0.9% NaCl saline at 640 μg/mL) was *i*.*v*. infused for 2 minutes at a dose of 10 µg/kg/minute (Dob10). Subjects were allowed to recover until heart rate stabilized. Then an additional 2-minute baseline was recorded before administering 20 µg/kg/minute *i*.*v*. infusion for two minutes (Dob20). Acquisition software (LabChart Pro version 7.3.2, AD Instruments) generated heart rate (BPM), peak maximum pressure slope (+dP/dT; mmHg/sec), and peak minimum pressure slope (−dP/dT; mmHg/sec), peak systolic pressure (mmHg), end diastolic pressure (mmHg), mean pressure (mmHg), and double product (systolic mmHg • BPM) from the LVP tracing. LVP data were noted at the end of each baseline period, at peak response for Dob10 and Dob20, and at two minutes of recovery for comparison across groups. One subject was lost in the DE150 group due to surgical complication, yielding n = 5; otherwise each group had n = 6.

### Serum collection from SHRs one-hour after exposure

SHRs from the Blood Collection cohort were exposed to FA, DE150, or DE500 (n = 6/group) and then anesthetized with sodium pentobarbital (100 mg/kg *i*.*p*.), one hour after exposure. Once unresponsive, SHRs underwent laparotomy and 7 to 10 mL of blood was collected from the abdominal aorta using a 20-gauge needle and syringe. Blood was centrifuged at 4 °C centrifuge for 10 minutes at 1500 rpm in serum separator tubes. Serum was then immediately collected, snap frozen, and stored at −80 °C until ready for *in vitro* experiments and proteomic assessment.

### Cytotoxicity assay in rat aortic endothelial cells treated with serum from exposed SHRs

Cytotoxicity was evaluated with a 96-well plate MTS assay (Promega, Madison, WI). To do so, RAECs (ATCC, Manassas, VA) were grown to 90% confluency in 96-well plates. Rat serum collected from the Blood Collection cohort, 1-hour after exposure was diluted to 10% in cell culture media and added to RAECs for 24 hours in triplicate (n = 6). After serum treatment, media was removed, and the MTS assay was performed. Absorbance was read at 490 nm on a spectrophotometer (BioTek Synergy HT, BioTek, Winooski, VT). Each sample triplicate was averaged, divided by the average absorbance from the FA group (n = 6), and multiplied by 100 to yield percent viability compared to FA (n = 6 data points/group).

### Nitric oxide synthase activity in rat aortic endothelial cells treated with serum from exposed SHRs

NOS activity was evaluated using a 96-well plate absorbance assay (#NB78, Oxford Biomedical Research®, Rochester Hills, MI). First, RAECs were grown to confluency in 24-well plates. Rat serum collected from the Blood Collection cohort 1-hour after exposure was diluted to 10% in cell culture media and added to RAECs for 24 hours in individual wells (n = 6). Following treatment, the media containing rat serum was removed. Cells were detached with 250 µL of trypsin. Trypsin was neutralized with 250 µL of media containing fetal bovine serum. Samples were transferred to 1.5 mL tubes and then centrifuged at 1200 rpm for 5 minutes. Media was removed. The cell pellet was washed with PBS and then centrifuged again at 1200 rpm for 5 minutes. PBS was removed. Lysis buffer (50 µL) was added to resuspend the cell pellet. A Pierce BCA Protein Assay was run to determine protein concentration of each sample. Protein from each sample (50 µg) was plated in duplicate (n = 6) into a 96-well format NOS assay. The assay was performed according to manufacturer’s instructions and the resulting absorbance was read at 540 nm on a spectrophotometer (BioTek Synergy HT, BioTek, Winooski, VT). The absorbance average from the assay blanks was subtracted from all samples and standards. The NO concentrations for each sample well were calculated based on the assay standard curve. All duplicate NO concentration values were averaged, divided by the average NO concentration value for the FA group (n = 6), and multiplied by 100 to yield percent NO produced compared to FA (n = 6 data points/group).

### RT-PCR of biomarkers in rat aortic endothelial cells treated with serum from exposed SHRs

Expression of Alox15, Tbxas, Edn1, Ho1, Il33, Nos3, Icam1, Vcam1, Cxcl2, and Il6 mRNA were assessed in RAECs treated for 24 hours with serum collected from exposed SHRs. See Supplemental Material for details.

### Assessment of 15-HETE in serum collected from exposed SHRs

15-HETE was extracted from serum samples and analyzed by ELISA (ADI-900–051, Enzo Life Sciences, Inc., Farmingdale, NY, USA). See Supplemental Material for details.

### Vascular cell adhesion molecule-1 expression in rat aortic endothelial cells treated with serum from exposed SHRs

VCAM-1 expression was determined by flow cytometry. First, RAECs were grown to 90% confluency in 96-well plates. Rat serum collected from the Blood Collection cohort, 1-hour after exposure was diluted to 10% in cell culture media and added to RAECs for 3 hours (n = 6/serum sample). Following the 3-hour treatment, cells were collected, washed with PBS, and fixed with 2% paraformaldehyde. After undergoing a PBS wash, cells were incubated for 30 min with a VCAM-1 antibody (1:200) (Cat# 13-1060-81, eBioscience, San Diego, CA) followed by the incubation for 30 min with FITC-labeled streptavidin (1:200) (Cat# 11-4317-87, eBioscience) at room temperature. Cells were then washed with PBS and resuspended in 200 µl prior to evaluation by flow cytometry (Accuri C6 Flow Cytometer, BD Biosciences, San Jose, CA). Control cells not exposed to rat serum were similarly processed but did not receive the primary VCAM-1 antibody, only the secondary. This sample was utilized to subtract background binding by the secondary antibody. 10,000 events were analyzed for each sample and measuring mean fluorescence. The mean fluorescence reported for the “no stain” (no primary) controls for each sample were subtracted from all mean fluorescence values for the stained samples. The resulting data values for each sample were divided by mean value for the FA group (n = 6) and then multiplied by 100 to yield percent VCAM-1 expression compared to FA (n = 6 data points/group).

### Sample preparation for serum proteomic analysis

For a more detailed description of proteomic assessments, see the Supplemental Materials. In short, rat serum that was collected from the Blood Collection Cohort, 1-hour after exposure and frozen. Prior to assessment, serum was thawed, diluted 5-fold and filtered at 0.22 µm to remove particulates. Samples were depleted of highly abundant proteins albumin, IgG, and transferrin using a Multiple Affinity Removal Column (Agilent Technologies, Santa Clara, CA, USA) to allow for quantification of the lower abundance proteins^63^. The resultant flow-through fraction was trypsin-digested overnight using a modified Filter Aided Sample Preparation method^62^. A Pierce™ BCA assay kit (Thermo Fisher Scientific, Waltham, MA, USA) was used per the manufacturer’s instructions for the enhanced assay to measure the protein concentration of each low-abundant protein fraction. Peptides were fractionated by high pH fractionation^64,65^ using an offline 1200 series Agilent HPLC system. The resulting fractions were analyzed by LCMS/MS on an Agilent 6520 QTOF. Data were collected in positive ion polarity over mass ranges 100–2200 m/z at a scan rate of 3 spectra/second.

### Serum proteomic analysis

Identified spectra were used to populate an accurate mass and time (AMRT) database. Unfractionated digested rat serum samples were analyzed by LCMS for differential analysis. Peptides which passed differential filters (ANOVA and fold change, see *Statistics* section) were identified in one of two ways: differential peptides were used to search the AMRT database. The remaining peptides without database hits were then targeted for by LCMS/MS and the resulting spectra were searched against the SwissProt *Rattus Norvegicus* database in Spectrum Mill (Agilent, Santa Clara, CA) allowing up to 2 missed tryptic cleavages with variable carbamidomethyl (C), deamidated (N), oxidation (M), N-term pryroglutamic acid (Q), and phosphorylated (STY) modifications. Data was extracted and aligned for mass and time using a recursive strategy performed in Profinder software (Agilent) and Mass Profiler Professional software (Agilent). Peptides were annotated using ID Browser software (Agilent) by matching mass and retention time from aligned experimental data to peptide entries in the AMRT library generated previously from MS/MS data. The annotated peptide list was filtered to peptides that were found in at least 4 of 6 samples and in at least 1 of 3 conditions. Peptides were rolled up into protein abundances using sort and subtotal functions within Microsoft Excel.

### Statistical analyses

Biological data are reported as mean ± SD (or SEM for clarity in Fig. 2) or presented as boxplots with all data points shown. Box edges identify the interquartile range, the middle line identifies the median, the “+” identifies the mean, and the whiskers identify the minimum and maximum data values. Data were analyzed and graphed with Graphpad Prism 6 software version 6.07 (La Jolla, CA, USA). In Fig. 2, LVP data are mean ± SEM for improved clarity (n = 6/group). These data were analyzed by two-way repeated measures ANOVA across exposure groups, with Tukey’s multiple comparisons tests and multiplicity adjusted *p*-values; *denotes *p* < *0*.*05* between FA and DE500; †denotes *p* < *0*.*05* between FA and DE150. In Figs 3 and 4, normalized LVP data and endothelial bioactivity data were analyzed by one-way ANOVA across exposure groups, with Tukey’s multiple comparisons test and multiplicity adjusted *p*-values, and by one-way ANOVA across exposure groups with linear trend analyses for multiple comparisons; *denotes *p* < *0*.*05* vs. FA; †denotes *p* < *0*.*05* vs. DE150; ‡denotes *p* < *0*.*05* for linear trend. In Table 2, animal weight data were analyzed by two-way ANOVA across cohort (LVP vs. Blood Collection) within exposure group, with Bonferroni’s multiple comparisons test and multiplicity adjusted *p*-values; data were also analyzed by two-way ANOVA across exposure group within cohort, with Tukey’s multiple comparisons test and multiplicity adjusted *p*-values. In Table 3, LVP data were analyzed by one-way ANOVA across exposure groups, with Tukey’s multiple comparisons test and multiplicity adjusted *p*-values: *denotes *p* < *0*.*05* vs. FA; †denotes *p* < *0*.*05* vs. DE150. To assess LVP parameter responsiveness and stability during dobutamine challenge and recovery, data were also analyzed by two-way repeated measures ANOVA across time point within each exposure group, with Tukey’s multiple comparisons test and multiplicity adjusted *p*-values: ↑denotes *p* < *0*.*05* for an increase from the previous time point; ↓denotes *p* < *0*.*05* for a decrease from the previous time point. Serum protein abundance values were imported into Mass Profiler Professional software (Agilent Technologies) for statistical analysis. In Table 4, protein significance was determined using a moderated t-test between combinations of the 3 groups: DE500 vs. FA, DE500 vs. DE150, or DE150 vs. FA when protein changes were 20% or greater. In Table 5, a one-way ANOVA with Tukey’s post-test was used on all 3 groups. Proteins were considered significant if changes produced *p* < *0*.*05*. Further details regarding statistical analysis of proteomic data can be found within the Supplemental Material. For Fig. 5, peptides that passed a one-way ANOVA significance test (*p* < *0*.*05*) with a Benjamini Hochburg multiple testing correction were clustered using unsupervised hierarchical clustering using the Euclidian distance metric and Ward’s linkage rule^66^.

## Supplementary information

Thompson et al Revised Supplemental Materials

Thompson et al Data File

## Data Availability

All data generated or analyzed during this study are included in this published article [and its Supplementary Information Files].
