# Supplementary material for: Early Proteome Shift and Serum Bioactivity Precede Diesel Exhaust-induced Impairment of Cardiovascular Recovery in Spontaneously Hypertensive Rats
Source: Sci Rep. 2019 May 3;9:6885. doi: 10.1038/s41598-019-43339-8 (PMC6499793; doi:10.1038/s41598-019-43339-8)
Supplement: Supplementary file 1 — Thompson et al Revised Supplemental Materials [file 41598_2019_43339_MOESM1_ESM.docx]

**Supplemental Materials for “Early Proteome Shift and Serum Bioactivity Precede Diesel Exhaust-induced Impairment of Cardiovascular Recovery in Spontaneously Hypertensive Rats”**

Leslie C. Thompson^1^* (thompson.leslie@epa.gov), Jonathan H. Shannahan^2^* (jshannah@purdue.edu), Christina M. Perez^3^ (cperez14@com.edu), Najwa Haykal-Coates^1^ (coates.najwa@epa.gov), Charly King^1^ (king.charly@epa.gov), Mehdi S. Hazari^1^ (hazari.mehdi@epa.gov), Jared M. Brown^4†^ (jared.brown@ucdenver.edu), and Aimen K. Farraj^1†^ (farraj.aimen@epa.gov)

*^†^These authors contributed equally

^1^Environmental Public Health Division, U.S. Environmental Protection Agency, Research Triangle Park, NC, USA; ^2^School of Health Sciences, Purdue University, West Lafayette, IN, USA; ^3^College of the Mainland, Texas City, TX, USA; ^4^Department of Pharmaceutical Sciences, University of Colorado Anschutz Medical Campus, Aurora, CO, USA.

†Corresponding Authors:

Dr. Aimen K. Farraj, Ph.D., DABT

109 T.W. Alexander Dr

Mail Code: B105-02

Research Triangle Park, NC 27709

[p] 919-541-5027

[f] 919-541-0034

[e] Farraj.aimen@epa.gov

Dr. Jared Brown, Ph.D.

Skaggs School of Pharmacy and Pharmaceutical Sciences

12850 E Montview Blvd

Aurora, CO 80045

[p] 303-724-8213

[f] 303-724-7266

[e] jared.brown@ucdenver.edu

**SUPPLEMENTAL METHODS**

*RT-PCR of Mediators of Inflammation in* *Rat Aortic Endothelial Cells Treated with Serum from Exposed Rats:* Expression of *Alox15*, *Tbxas*, *Edn1*, *Ho1*, *Il33*, *Nos3*, *Icam1*, *Vcam1*, *Cxcl2*, and *Il6* mRNA were assessed in RAECs treated with serum collected from exposed rats. RAECs were plated in duplicate and treated with 10% serum collected from exposed rats (n = 6) in 24-well plates. After the 24-hour treatment period RAECs, total RNA was isolated from cells using Direct-zol RNA MiniPrep (Zymo Research Corp., Irvine, CA) via manufacturer instructions and quantified by nanodrop (Nanodrop 2000c Spectrophotometer, Thermo Scientific). Total RNA was reverse transcribed to cDNA using an iScript cDNA Synthesis Kit (Bio-Rad Laboratories, Hercules, CA). Quantitative real-time PCR was performed for Vcam-1, Alox-15, Tbxas, Edn-1, Ho-1, Il-33, Nos3, Icam1, Cxcl-2, Il-6 and GAPDH (control) using SsoAdvancedTM SYBR Green Supermix (Bio-Rad) and QuantiTect primer assays (Qiagen, Balencia, CA). Relative mRNA fold changes were calculated considering serum-free media exposed cells as control and normalized to GAPDH as the internal reference.

*Assessment of 15-HETE in Serum Collected from Exposed Rats:* Serum concentration of 15-HETE was assessed with a commercially available 96-well ELISA (ADI-900-051, Enzo Life Sciences, Inc., Farmingdale, NY, USA). In short, serum samples were thawed on ice, diluted 1:50 with assay buffer, and incubated with assay conjugate and antibody overnight at 4°C. The next day, samples and standards were incubated with assay substrate solution for 1 hour at 37°C. After adding assay stop solution, absorbance data was collected immediately at 405 nm. Samples were run in duplicate at n = 6.

DETAILED DESCRIPTION OF PROTEOMICS

*Immunodepletion:* In serum and plasma, the presence of high abundance proteins (*e.g.* albumin, IgG, transferrin) can impede the identification and quantification of lower abundant proteins [1]. A mouse Multiple Affinity Removal column (MARS Ms-3, Agilent Technologies) was used to remove the top 3 most abundant proteins: albumin, IgG and transferrin, from rat serum collected from the Blood Collection cohort, 1-hour after exposure. For this purpose, 20 µL of rat serum was diluted five-fold with MARS-A buffer (Agilent Technologies) and filtered through a 0.22 µm cellulose acetate spin filter (Agilent Technologies) by centrifuging at 18,000 x g for 6 minutes to remove particulate matter. An Agilent 1200 series HPLC system, which consists of a quaternary pump, high performance autosampler (HiP ALS) and DAD detector, coupled with a fraction collector were used to perform the immunodepletion. For each sample, 13 µL of serum was injected onto the MARS Ms-3 column. A gradient of 100% MARS-A buffer for 9 minutes at 0.25 mL/minute was used to bind high abundant proteins and elute and collect flow-through containing low abundant proteins. Approximately 1 mL of flow-through was collected. The mobile phase composition was switched to 100% MARS-B buffer from 9.01 min to 12.5 minutes at a flow rate of 1.0 mL/minute to elute bound high abundant proteins; these were not collected. The column was re-equilibrated by switching the mobile phase composition to 100% MARS-A buffer at 1.0 mL/minute for an additional 10.5 minutes before another injection cycle was performed.

*Tryptic Digestion:* The resultant flow-through fraction was trypsin-digested overnight using a modified Filter Aided Sample Prep (FASP) method [2]. A BCA assay kit (Pierce) was used per the manufacturer’s instructions for the enhanced assay to measure the protein concentration of each low-abundant protein fraction. Then a total of 63.8 µL of a 10% SDS in 100 mM Tris-HCl pH 8.5 (Lysis Buffer) solution and 10.73 µL of 1M TCEP was added to 1 mL of sample. The sample was heated at 95°C for 10 minutes and allowed to cool at room temperature before probe sonicating 3 times at 5 watts in 2-3 second bursts. After sonication, the volume of solution was measured and 451.9 mg of urea was added to produce an 8M solution. Sulfahydryls were alkylated and reduced by adding IAA to a final 25 mM concentration. Sample was incubated in the dark for 30 minutes at room temperature with gentle shaking. A 30 kD Molecular Weight Cut-Off spin filter (30kD MWCO filter, Amicon) was pre-washed with 300 µL of 8M urea solution by gently vortexing and then centrifuging at 14000 x g for 5 minutes at room temperature. Residual 8M urea was discarded by inverting the spin filter and centrifuging at 1000 x g for 30 seconds. In the remaining steps the flow-through was discarded after each spin and all centrifuge steps were conducted at 14000 x g for 6 minutes at room temperature. The denatured, reduced and alkylated sample was added to a prewashed spin filter and centrifuged to concentrate the solution to a final volume of approximately 90 µL. After sample concentration, samples were washed with 300 µL of the following solutions 3 times in the listed order: 8M urea, 2M urea, and 5% TFE in 50 mM ammonium bicarbonate (5% TFE). 100 µL of 5% TFE was added to the washed sample in the spin filter and was transferred to a fresh collection tube. To each sample, 1:50 enzyme to protein of lysyl endopeptidase (Lys-C) was added and samples were shaken gently on a vortexer for 4 hours at room temperature before 1:40 enzyme:protein of trypsin was added and samples were incubated overnight at 37°C. Digested peptides were eluted off the spin filter by centrifuging at 14000 x g for 10 minutes at room temperature. Eighty µL of 5% TFE was added to the spin filter to recover more peptides by gently vortexing and centrifuging at 14000 x g for 10 minutes at room temperature. This step was repeated once more. Approximately 200 µL of flow-through containing digested peptides was collected. An aliquot of 65 µL was pooled from each sample and dried down for high pH fractionation; the remainder was transferred to a 1.5 mL micro-centrifuge tube and dried down in a speedvac at room temperature for 3 hours. Dried samples were resuspended in 50 µl of 3% ACN/0.1% formic acid, transferred to auto sampler vials, and analyzed by mass spectrometry.

*High pH Proteome Fractionation:* Although the top 3 most abundant proteins were depleted, the dynamic range of protein concentration in serum is still multiple orders of magnitude, which limits the amount of proteins and peptides that can be identified [3, 4]. High pH peptide fractionation is a technique used to simplify complex samples and thereby reduce the dynamic range and maximize our chances of identifying more proteins. High pH fractionation was performed using an offline 1200 series Agilent HPLC system, which consists of a capillary pump, micro WPS autosampler, thermostat column compartment and DAD detector, equipped with a fraction collector. Peptides were separated on a X-Bridge Peptide BEH C18 130A, 3.5 µm, 1 mm x 150 mm Column (Waters) using two mobile phases: Mobile phase A was aqueous 10 mM ammonium formate adjusted to pH 10 with ammonium hydroxide; Mobile phase B was 90% acetonitrile 10 mM ammonium formate adjusted to pH 10 with ammonium hydroxide. Peptides were loaded onto the column at 3% B for 4 minutes flowing at 80 µL/minute. The majority of peptides were resolved from 4 minutes to 66 minutes when mobile phase B was steadily increased from 3% to 45%. Mobile phase B was increased to 70% over the next 4 minutes and held for 10 minutes to remove any remaining hydrophobic compounds bound to the column. The column was re-equilibrated for 20 minutes at 3% B. The pooled sample for high pH fractionation was re-suspended in 90 µL of 3% acetonitrile in 10 mM ammonium formate adjusted to pH 10 with ammonium hydroxide. Triplicate 25 µL injections (~235 µg each) and 45 fractions were collected. The first 2 fractions were collected for 4 minutes each, the next 40 were collected 1.125 minutes each and the last 3 fractions were collected 2.33 minutes each. In all, eluent was collected from 0 to 60 minutes. Signal was collected at a wavelength of 214 nm. Each fraction was individually dried down in a speedvac at 45°C for 3 hours. Each fraction was re-suspended in 25 µL of 3% ACN in 0.1% formic acid prior to LCMS analysis.

*Accurate Mass and Retention Time Library:* High pH fractions were loaded onto a capillary ProntoSIL C18AQ trap (Nano LCMS Solutions, PN-41002) and resolved on-line using a 0.3 x 150 mm, 3.0µ 200A ProntoSIL C18AQ reverse phase capillary column (Nano LCMS Solutions, PN-31005) using a 1260 series HPLC (Agilent). Mobile phases consisted of water + 0.1% formic acid (A) and 90% acetonitrile + 0.1% formic acid (B). Sample was loaded onto the trapping column at 10 µL/min for 2 minutes at initial buffer conditions before being chromatographically separated at a flow rate of 5 µL/min using a gradient of 3-45% B from 4 to 64 minutes. The gradient was followed by a column wash at 80% B for 3 minutes before re-equilibration at initial conditions for 10 minutes. Data was collected on 6520 Q-TOF equipped with a capillary-flow nebulizer (Agilent) operated using intensity-dependent CID MS/MS. The capillary voltage, nebulizer pressure, drying gas flow, and drying gas temperature were set to 4000 V, 40 psig, 6.0 l/min and 325C, respectively. Data was collected in positive ion polarity over mass ranges 100– 2200 m/z at a scan rate of 3 spectra/sec. All charge states were allowed except singly charged species were excluded from being selected during MS/MS acquisition and charge states 2 and 3 were given preference. Spectrum Mill software (Agilent) was used to extract, search, and summarize peptide identity results. Spectra were searched against the SwissProt Rattus Norvegicus database allowing up to 2 missed tryptic cleavages with variable carbamidomethyl (C), Deamidated (N), Oxidation (M), N-term Pyroglutamic acid (Q), and phosphorylated (STY) modifications. Spectra that were not matched were subsequently searched against the SwissProt Rattus Norvegicus database allowing up to 2 missed tryptic cleavages with semi-tryptic enzyme setting with no variable modifications allowed. The monoisotopic peptide mass tolerance allowed was ± 20.0 ppm and the MS/MS tolerance was ± 50 ppm. A minimum peptide score of 8, scored peak intensity of 50%, and protein score of 13 were used as cut-offs for the generation of an AMRT library. Digested low abundant proteins were acquired using the same method parameters as the high pH fractionation library samples except samples were run in MS-only mode at a scan rate of 1.5 spectra/s.

*Proteomic Data Analysis:* Data was extracted and aligned for mass and time using a recursive strategy performed in Profinder software (Agilent Technologies) and Mass Profiler Professional software (Agilent Technologies). First, peptide molecular features were extracted in Profinder using the Find by Molecular Feature algorithm. An ion count threshold of 1000 counts, an absolute chromatographic peptide peak height threshold of 5000 counts, and a quality score threshold of 90 were applied. Two or more ions were required for each peptide. Charge states 1-5 were included with H^+^ and Na^+^ allowed as charge carriers. Retention time and mass window alignment tolerances were set to 0.5 min. and 15 ppm, respectively. The extracted peptide compound list was exported to MPP and filtered so that only compounds found in at least 2 of 18 samples were retained. This list was imported into Profinder and used as a target list for targeted feature extraction using the Find by Ion algorithm. Charge states, adducts, isotope model, retention time window, and mass window alignment settings were set to the same values used with the molecular feature extractor. An absolute chromatographic peak height threshold of 4000 counts was applied and only compounds that had a Find by Ion score greater than 50 were retained. Final extraction results were exported to MPP for differential analysis.

Peptides were annotated using ID Browser software (Agilent Technologies) by matching mass and retention time from aligned experimental data to peptide entries in the AMRT library generated previously from MS/MS data. Match tolerance windows of 15 ppm and 0.4 min RT were allowed. Annotations with a match score greater than 50 and mass ppm error less than ±10 ppm were retained. Match scores are based on calculated neutral mass, isotope m/z, isotope peak heights, and retention time tolerance. The annotated peptide list was filtered to peptides that were found in at least 4 of 6 samples, in at least 1 of 3 conditions. Peptide annotations and normalized log2 transformed values on a per sample basis were exported to a spreadsheet where peptides were rolled up into protein abundances using sort and subtotal functions. Briefly, Log2 transformed data was transformed back into raw data values and summed on a per protein and sample basis, *e.g.* if there were 6 unique peptide matches that corresponded to a single protein ID then all 6 raw peptide abundances were summed to generate an overall protein abundance value on a per sample basis. Imputation was not performed. Protein abundance values were imported into MPP for statistical analysis. Protein significance was determined using an ANOVA on all three sample types or using a moderated t-test between combinations of the three sample types: High Diesel vs. Air, High Diesel vs. Low Diesel or Low Diesel vs. Air. Proteins were considered significant if they had a *p*-value < 0.05.

**SUPPLEMENTAL DATA**

*mRNA Expression Data:* No statistically significant differences were found in *Alox15*, *Tbxas*, *Edn1*, *Ho1*, *Il33*, *Nos3*, *Icam1*, *Vcam1*, *Cxcl2*, and *Il6* mRNA expression in RAECs after 24-hour treatment with serum collected from exposed rats (see Supplemental Table S1 below). However, as shown in Supplemental Figure S1, Alox15 expression was > 2-fold downregulated relative to Alox15/Gapdh ratio in the FA and DE150 groups.

*Serum 15-HETE:* As shown in Supplemental Figure S1, no differences were found in serum 15-HETE.

**Supplemental Table S1** – *mRNA Expression presented as Fold Change from FA ± SD*

|  | Mean Fold Change from FA ± SD (N) | |
| --- | --- | --- |
| Target | DE150 | DE500 |
| *Alox15* | 1.3 ± 2.1 (4) | -2.2 ± 2.1 (6) |
| *Tbax* | 0.7 ± 1.4 (6) | 0.8 ± 1.4 (6) |
| *Edn1* | -0.1 ± 1.4 (6) | 0.5 ± 1.4 (6) |
| *Ho1* | 0.4 ± 1.2 (6) | 0.0 ± 1.2 (6) |
| *Il33* | -0.9 ± 2.1 (5) | -0.9 ± 1.7 (6) |
| *Nos3* | -0.4 ± 1.1 (6) | -0.9 ± 1.0 (6) |
| *Icam1* | 1.2 ± 1.2 (6) | 0.3 ± 1.5 (6) |
| *Vcam1* | 0.5 ± 1.6 (6) | -1.0 ± 1.9 (6) |
| *Cxcl2* | 1.2 ± 0.2 (6) | -1.0 ± 1.2 (6) |
| *Il6* | -0.7 ± 1.8 (6) | -0.8 ± 1.6 (6) |

**

**

**Supplemental Figure S1 – *Alox15* mRNA expression and serum 15-HETE.** We tested *Alox15* expression based on the findings of the multiethnic study of atherosclerosis (MESA) that showed polymorphisms in *ALOX15* correlating with proximity to major roadways [5]. In panel A, we found that expression of *Alox15* mRNA was down by > 2-fold on average, though not statistically different than expression in FA or DE150 groups. Two data points were considered outliers and removed by Iterative Grubbs (Alpha = 0.05) based on fold change data from all samples including FA group. Next, we assessed serum 15-HETE in serum samples to test the possibility the Alox15 was being down-regulated in a negative feedback mechanism. Panel B shows no evidence of differences in 15-HETE in serum collected from rats one hour after exposure. **REFERENCES**

1. Legg KM, Powell R, Reisdorph N, Reisdorph R, Danielson PB. 2014. Discovery of highly specific protein markers for the identification of biological stains. *Electrophoresis* 35:3069-3078.
2. Wisniewski JR, Zougman A, Nagaraj N, Mann M. 2009. Universal sample preparation method for proteome analysis. *Nat Methods* 6:359-362.
3. Anderson NL, Anderson NG. 2002. The human plasma proteome: History, character, and diagnostic prospects. *Mol Cell Proteomics* 1:845-867.
4. Issaq HJ, Xiao Z, Veenstra TD. 2007. Serum and plasma proteomics. *Chem Rev* 107:3601-3620.
5. Van Hee VC, Adar SD, Szpiro AA, Barr RG, Diez Roux A, Bluemke DA, et al. 2010. Common genetic variation, residential proximity to traffic exposure, and left ventricular mass: The multi-ethnic study of atherosclerosis. *Environ Health Perspect* 118:962-969.
